# Supplementary material for: DSC, FT-IR and NIR with Chemometric Assessment Using PCA and HCA for Estimation of the Chemical Stability of Oral Antidiabetic Drug Linagliptin in the Presence of Pharmaceutical Excipients
Source: Molecules. 2022 Jul 3;27(13):4283. doi: 10.3390/molecules27134283 (PMC9268681; doi:10.3390/molecules27134283)
Supplement: Supplementary file 1 [file molecules-27-04283-s001.zip › molecules-1780701-supplementary.pdf]

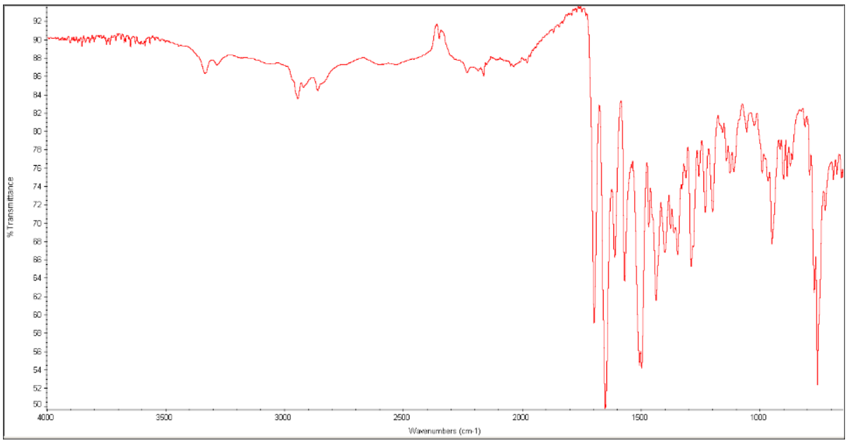

a

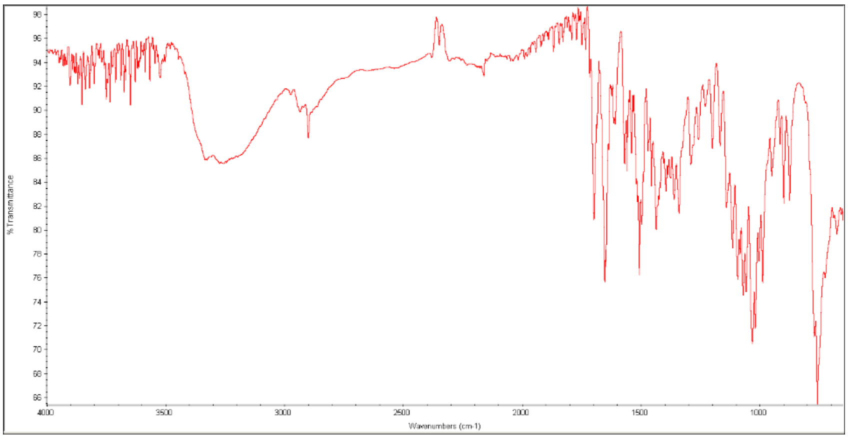

b

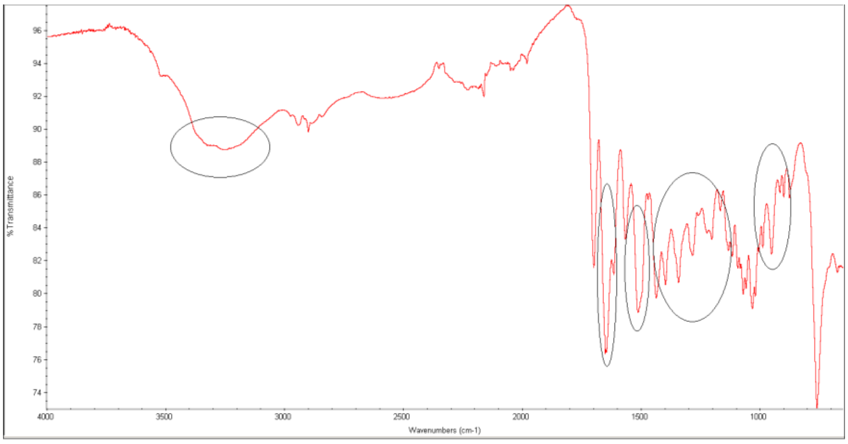

c

Figure S1. a) pure LINA, b) LINA+LAC, c) LINA+LAC (stressed).

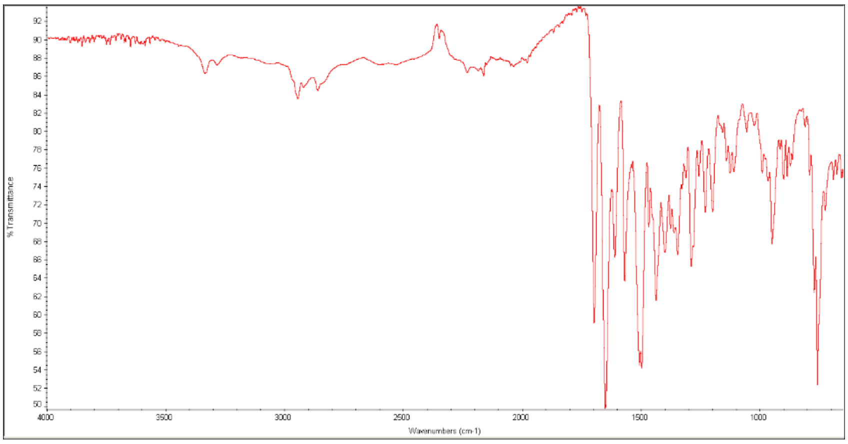

a

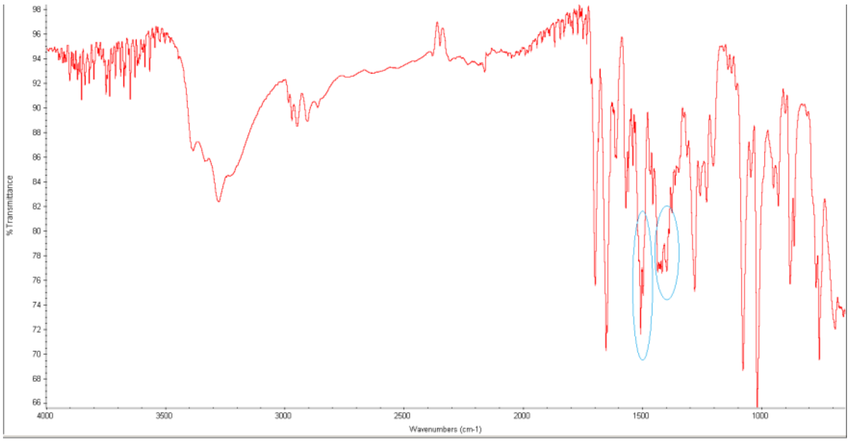

b

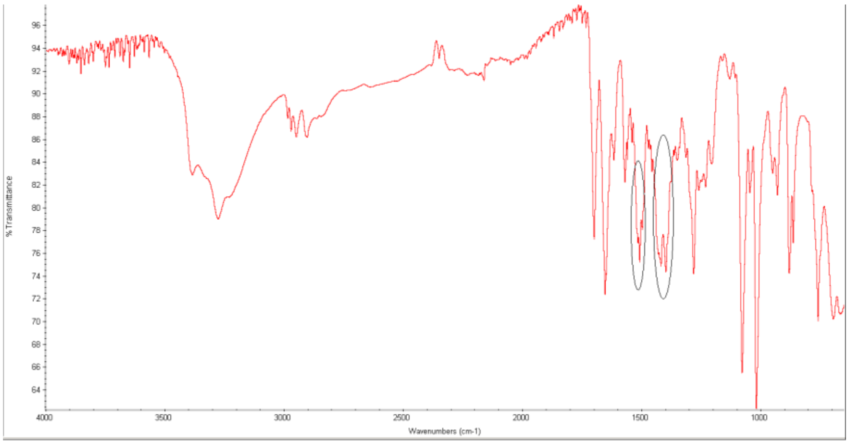

c

Figure S2. a) pure LINA, b) LINA+MAN, c) LINA+MAN (stressed).

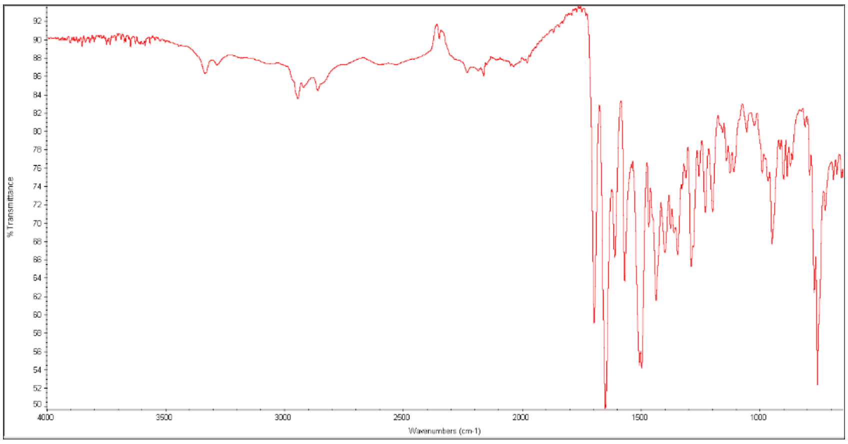

a

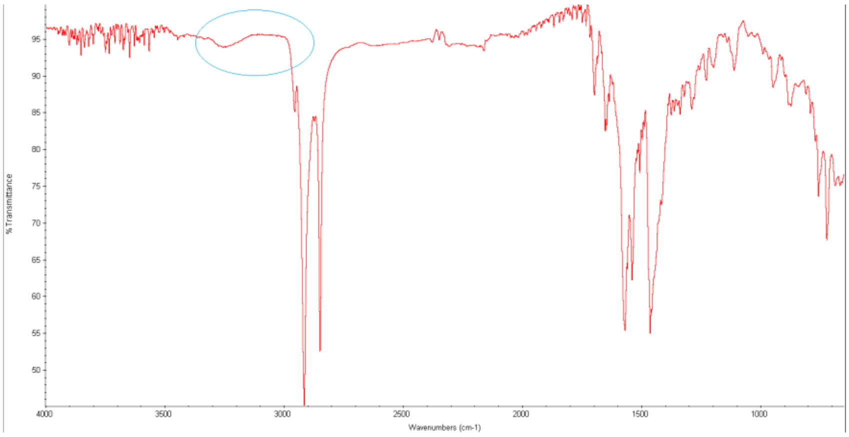

b

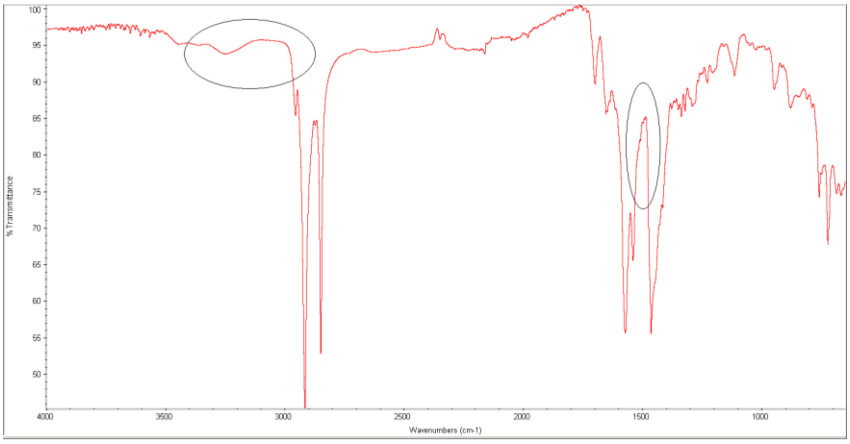

c

Figure S3. a) pure LINA, b) LINA+MGS, c) LINA+MGS (stressed).

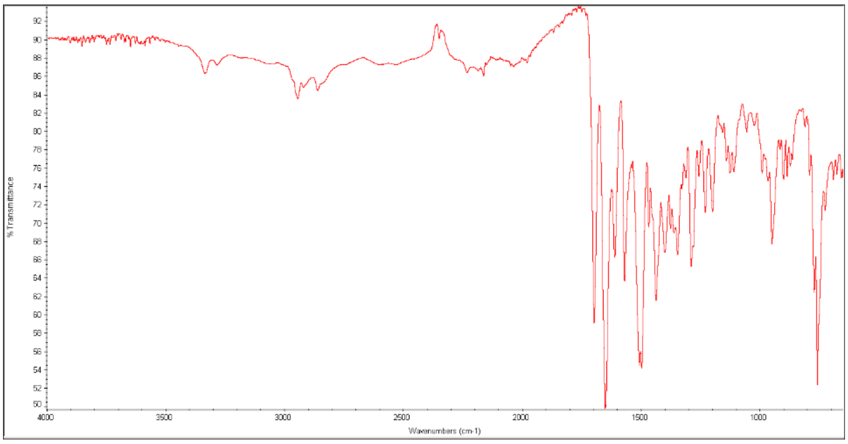
a

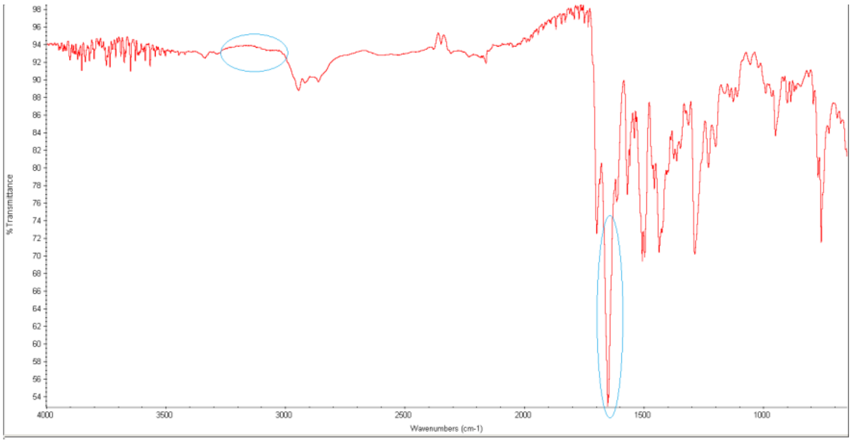
b

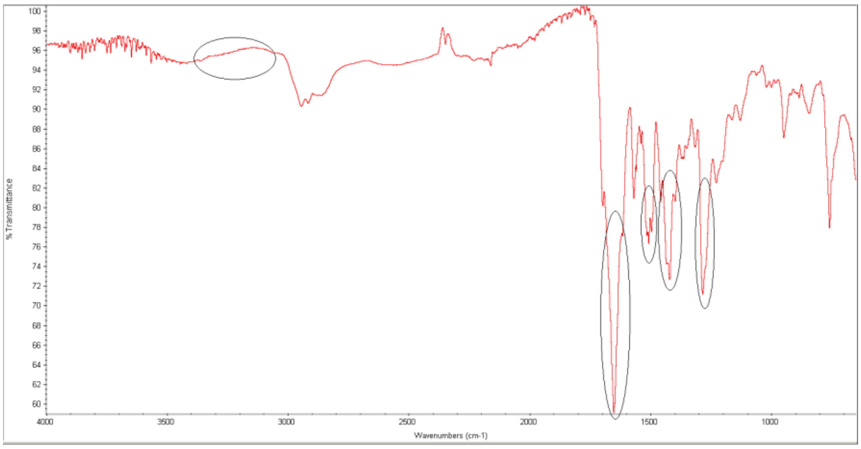
c

Figure S4. a) pure LINA, b) LINA+PVP, c) LINA+PVP (stressed).
